# Healthy Dietary Patterns with and without Meat Improved Cardiometabolic Disease Risk Factors in Adults: A Randomized Crossover Controlled Feeding Trial

**DOI:** 10.3390/nu16152542

**Published:** 2024-08-03

**Authors:** Erica R Hill, Yu Wang, Eric M Davis, Wayne W Campbell

**Affiliations:** Department of Nutrition Science, Purdue University, West Lafayette, IN 47907, USA

**Keywords:** healthy eating pattern, lean beef, omnivore, vegetarian, lipids, lipoproteins, blood glucose, insulin, cardiovascular disease, type 2 diabetes mellitus

## Abstract

We assessed the effects of consuming a U.S.-style healthy dietary pattern (HDP) with lean, unprocessed beef (BEEF) compared to a U.S.-style HDP without meat (vegetarian, VEG) on short-term changes in cardiometabolic disease (CMD) risk factors in adults classified as overweight or obese. Forty-one adults (22 females, 19 males; age 39.9 ± 8.0 y; BMI 29.6 ± 3.3 kg/m^2^; mean ± SD) completed two 5-week controlled feeding periods (randomized, crossover, controlled trial). For the BEEF HDP, two 3-oz (168-g) servings/d of lean, unprocessed beef were predominately substituted for some starchy vegetables and refined grains in the VEG HDP. Baseline and post-intervention measurements were fasting CMD risk factors, with serum low-density lipoprotein (LDL), total cholesterol (TC), and total apolipoprotein B as primary outcomes. VEG reduced LDL, insulin, and glucose compared to BEEF. Reductions did not differ between VEG vs. BEEF for TC, high-density lipoprotein (HDL), apolipoprotein A1, small, dense LDL IV, buoyant HDL2b, TC-to-HDL ratio, and systolic blood pressure. Total apolipoprotein B and all other CMD risk factors measured were not influenced by HDP type nor changed over time. Adopting a U.S.-style HDP that is either vegetarian or omnivorous with beef improved multiple cardiometabolic disease risk factors among adults classified as overweight or obese.

## 1. Introduction

High red meat consumption is one of the hallmark characteristics of a Western-style dietary pattern [1]. Red meat within this dietary pattern is associated with an increased risk for cardiometabolic diseases (CMD), including cardiovascular disease (CVD) and type 2 diabetes mellitus (T2DM) [2,3]. As a result, the U.S. government and public health organizations [4,5,6] recommend consuming healthy dietary patterns (HDPs) with an emphasis on increasing plant-based foods. This inadvertently encourages consumers to avoid red meat intake. The hesitancy to reduce or stop consuming red meat is one hindrance to U.S. residents adopting and maintaining HDPs [7].

Lean, unprocessed beef is a notable source of monounsaturated fatty acids and is low in sodium, both of which are shown to have favorable effects on CMD risk factors, especially when consumed in the context of a HDP [8,9]. Results from experimental randomized controlled feeding trials (RCTs) that assessed lean and unprocessed beef within an HDP suggest beneficial or neutral effects on CMD risk factors [8,9,10,11]. Findings from an RCT indicated greater improvements in lipid and lipoprotein risk factors in an euenergetic (energy intake matching energy expenditure) HDP with higher lean beef intake (153 g/d) compared to low lean beef intake (20 g/d) [8]. Similarly, results from another RCT indicated middle-aged adults who consumed a euenergetic Mediterranean-style HDP with an average of 71 g/d of lean, unprocessed red meat, compared to 29 g/d, had greater reductions in serum total cholesterol and low-density lipoprotein cholesterol (LDL) [9]. These findings [8,9] suggested that HDPs that include higher amounts of lean, unprocessed red meat may promote improvements in some cardiometabolic disease risk factors in adults. 

Previous experimental research [8,9,10,11] predominately assessed different quantities of red meat or substituted red meat with other animal-based protein sources in the context of HDPs. Fewer studies assessed the impact of including red meat within a plant-based dietary pattern void of other flesh foods, i.e., a lacto-ovo vegetarian diet. Two studies comparing a red meat-containing diet intervention to a vegetarian diet intervention found no difference between the post-intervention diets in total and LDL cholesterol and triglycerides [12,13]. The results from RCTs with higher compared to lower lean, unprocessed red meat intakes, which included improving concentrations of apolipoprotein B along with other CVD risk factors [8,9,10,11], prompted the question of whether similar results would occur when comparing a VEG HDP to an omnivorous HDP with lean, unprocessed beef as the only source of meat/animal-based food. 

The primary purpose of this crossover RCT with all foods provided is to assess the effects of adults classified as overweight or obese consuming an omnivorous U.S.-style HDP with lean, unprocessed beef (BEEF, experimental) compared to a U.S.-style HDP without meat (VEG, control) on short-term changes in CMD risk factors. We hypothesized that consuming the BEEF HDP, compared to the VEG HDP, would enhance improvements in CMD risk factors, with serum LDL, total cholesterol (TC), and total apolipoprotein B being the primary outcomes. 

## 2. Materials and Methods

### 2.1. Experimental Design

The 16-week crossover RCT protocol, with investigator blinding, included one week of baseline testing (without dietary control), followed by two five-week long controlled feeding periods (either VEG HDP or BEEF HDP in random order), separated by participants consuming an unrestricted, self-selected dietary pattern (washout) for five weeks, where no food or beverages were provided to participants by study staff. Outcome measurements were obtained on standardized testing days, pre- and post-intervention. Primary outcomes are fasting serum LDL, total cholesterol, and apolipoprotein B; secondary outcomes are fasting serum triglycerides, HDL, lipoprotein particle sizes, insulin, glucose, HOMA-IR, systolic and diastolic blood pressure, waist circumference, hip circumference, and sagittal abdominal diameter; exploratory outcomes are participant adherence to and satisfaction with the two HDPs. 

### 2.2. Participant Inclusion Criteria

Participant inclusion criteria were male and female adults between the ages of 30–69 y, without diagnosed disease (non-diabetic, no acute illness, no history of cardiovascular events or liver or kidney dysfunction) who were classified as overweight or obese (BMI range: 25.0–37.0 kg/m^2^). Additional criteria were fasting serum total cholesterol < 260 mg/dL, LDL < 190 mg/dL, systolic and diastolic blood pressures (SBP/DBP) < 140/90 mmHg, body weight stable (±3 kg for previous 3 months), medication use consistent for previous 6 months, non-smoking, non-pregnant or lactating, physical activity regimen stable for previous 3 months, not lactose intolerant, and willing and able to consume the provided foods (VEG and BEEF HDPs). The participants were recruited from the Greater Lafayette, Indiana, U.S.A. area. A physician reviewed participant-provided medical history information and blood clinical chemistry, blood pressure, body weight, and height measurements to ensure they met the inclusion criteria. A member of the research team not involved with data collection or analysis randomly assigned participants to their intervention sequence. Randomization was achieved using an online randomization plan generator (http://www.randomization.com/) accessed on 1 June 2019. The randomization code was concealed until all participants’ testing and analyses of a priori independent outcomes were completed. This trial is registered at ClinicalTrials.gov (identifier: NCT03925142).

### 2.3. Ethics

The study protocol and documents were reviewed and approved by the Purdue University Biomedical Institutional Review Board (protocol #1809021091), with initial approval on 4 April 2019. All participants provided written informed consent before entry into the study and received monetary compensation for their participation.

### 2.4. Baseline Dietary Assessment

Self-chosen dietary intake data were collected and analyzed using the Automated Self-Administered 24 h (ASA24) Dietary Assessment Tool, version (2018), developed by the National Cancer Institute, Bethesda, MD [14,15]. Participants were asked to complete the dietary intake assessments on three non-consecutive days, with two weekdays and one weekend day, at baseline and during washout.

### 2.5. Dietary Interventions

A registered dietitian developed the menus using ProNutra software version 3.3 (Viocare, Inc., Princeton, NJ, USA). Participants’ total energy expenditure (requirement) was estimated using sex-specific equations for males and females ages 19 and older, classified as overweight or obese with low active physical activity, published by the Institute of Medicine [16], and menus were designed to maintain participants’ bodyweight throughout the study period. All protocol foods and beverages were procured, prepared, portioned, and provided to participants by staff members at the NIH-supported Indiana Clinical Research Center Bionutrition Facility at Purdue University. 

Lean meat is defined by the United States Department of Agriculture (USDA) as containing < 10 g total fat, <4.5 g saturated fat, and <95 mg cholesterol [17]. Unprocessed meat is defined as meat not preserved by smoking, curing, salting, and/or the addition of chemical preservatives [4]. Starchy vegetables are defined by the USDA as all fresh, frozen, and canned starchy vegetables: for example, white potatoes, corn, green peas, green lima beans, plantains, and cassava [18]. Refined grains, as defined by the USDA, are any grains and grain products with the bran and germ removed; any grain product that is not a whole-grain product [18]. 

The VEG and BEEF HDPs differed predominately in the amounts of lean, unprocessed beef, starchy vegetables, and refined grains (Table 1 and Table S1). We chose this approach because, like red meat, refined grains and starchy vegetables are generally recommended to be consumed less frequently as part of a HDP [4,18]. The BEEF HDP included 168 g/d (two 3-oz servings/d) of lean, unprocessed beef. To match the energy content between the BEEF and VEG HDPs, further adjustments were made by manipulating mainly dairy and fat intakes. In the BEEF dietary pattern, various cuts of lean, unprocessed beef were incorporated into mixed heterogenous dishes and included >85% lean ground beef, top sirloin, and beef top round [19]. 

Participants were weighed once weekly, and daily menu check-off lists were given to participants and returned twice per week to document self-reported deviations (additions, subtractions, or substitutions) from consuming the provided foods. Dietary adherence was assessed by documenting any deviations from the prescribed dietary intervention based on the returned menu check-off lists.

### 2.6. Clinical Assessments

Participants came in for testing twice the week before each intervention and twice during the last week of each intervention, for a total of eight standardized testing visits. Prior to all testing visits, participants were instructed to fast (no eating or drinking except water) for 10 h. Upon arrival at the clinical research facility, participants were seated in a reclined chair in a quiet, dimly lit room to rest for 15 min. Two systolic and diastolic blood pressure measurements were recorded and averaged. Bodyweight was measured, with participants wearing lightweight clothes. Waist circumference, hip circumference, and sagittal abdominal diameter were measured in triplicate using standardized protocols, with values averaged. 

Fasting blood samples were collected from the subject’s antecubital vein. The collection tubes contained either a clot activator to obtain serum or an anticoagulant agent (i.e., EDTA) to obtain plasma. Serum samples were held at room temperature, and plasma samples were refrigerated for 15 min before centrifugation. Fresh serum samples were sent to SpectraCell (Houston, TX, USA) to measure lipids, lipoprotein particle numbers and sizes, and markers of vascular inflammation, and to Mid America Clinical Laboratories (MACL) (Secaucus, NJ, USA) for a comprehensive metabolic panel. The serum samples sent to SpectraCell were centrifuged for 15 min at 4 °C and 3000 rpm, while the rest of the serum samples, including those sent to MACL, were centrifuged for 15 min at 4 °C at 4000× *g*. 

### 2.7. Dietary Satisfaction

Participants completed a dietary satisfaction questionnaire after each 5-week dietary intervention ended. There were 23 questions using a Likert scale, with responses ranging from ‘Strongly Disagree’ to ‘Strongly Agree’ (Table S2). The questionnaire was developed by combining features from two novel palatability and feasibility questionnaires used in similar studies [20,21] but was not explicitly validated as a dietary satisfaction measurement tool. 

### 2.8. Statistics

Power calculations indicated that 40 participants would provide ~95% power to detect differential changes between the control and intervention dietary patterns for LDL cholesterol (effect size = 0.58). Forty participants would also provide ~85% and ~90% power to detect differential changes between the control and intervention dietary patterns for total cholesterol (effect size = 0.48) and total apolipoprotein B (effect size = 0.54), respectively. All power calculations were two-tailed and based on results reported in a previous research study [9]. 

Two researchers independently entered all the data, which the study manager cross-checked for accuracy. Data from 41 participants who completed both dietary interventions were analyzed in SAS version 9.4 (SAS Institute, Cary, NC, USA) using the PROC MIXED procedure to compute a double repeated measures ANOVA. This procedure measured the (1) main effects of time (pre compared to post measurements; one-tailed), (2) interaction of time and intervention; (VEG changes compared to BEEF changes; two-tailed), (3) changes over time within VEG and within BEEF (intervention-specific effect indicated by time×ntervention *p* value < 0.05; one-tailed), (4) comparison of VEG and BEEF baseline measurements (intervention×time sliced by time; two-tailed), (5) comparison of BEEF and VEG post-intervention measurements (intervention×time sliced by time; two-tailed), and (6) comparison of baseline 1 and baseline 2 (before first and second intervention) measurements (trial×time interaction sliced by time; two-tailed) to determine if participants’ baseline 1 health status was re-established at baseline 2. At each time point, all outcome variables were controlled for age, sex, and body mass as appropriate. Body mass and composition were controlled for age and sex. Results are presented as adjusted least squares means ± standard error (SE), unless otherwise specified. Significance was set at *p* < 0.05, and all *p*-values were Tukey–Kramer adjusted for multiple comparisons. Dietary satisfaction data were analyzed in SPSS (version 28.0.0.0) using the “paired samples *t*-test” function. The two-tailed *p*-value was used to indicate significance (*p* < 0.05). 

## 3. Results

### 3.1. Subject Characteristics

**Figure 1:**
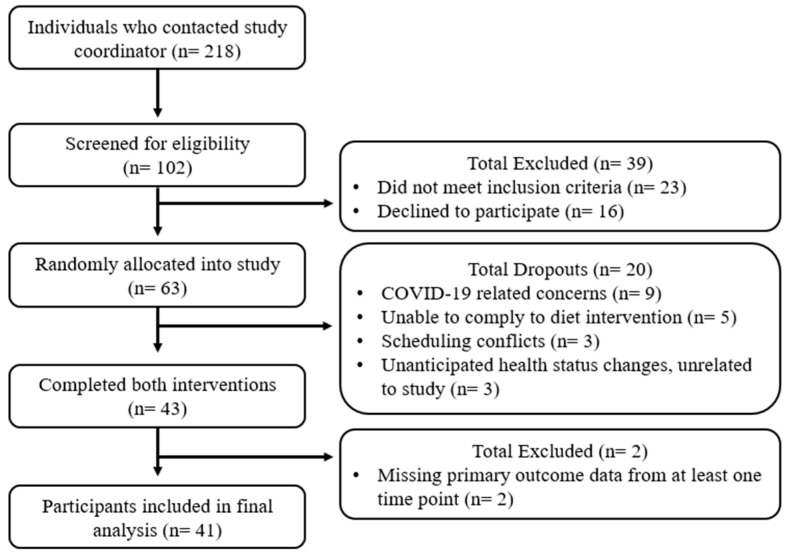
Study recruitment flow diagram.

Sixty-three participants were admitted into the study, with 20 dropping out for various reasons (i.e., disruptions from COVID-19, not adhering with the dietary interventions, scheduling conflicts, or unanticipated health status changes unrelated to the study). Forty-three participants (24 female and 19 male) completed both interventions, but two female participants were removed from the final analysis due to incomplete data. Data from 41 participants (22 female and 19 male) were included in the final analysis. See Figure 1 for the flow diagram and Table 2 for mean baseline 1 characteristics. 

### 3.2. Dietary Adherence and Satisfaction

Dietary adherence averaged 89% for each intervention and did not differ between the VEG and BEEF interventions (*p* > 0.05). Consistent with higher total protein intake [22], blood urea nitrogen was higher after the BEEF compared to the VEG dietary intervention (14.8 ± 0.5 vs. 12.8 ± 0.5 mg/dL, respectively, intervention × time (I×T) *p* = 0.001). 

The participants total diet satisfaction did not differ after consuming the VEG or the BEEF dietary interventions (*p* > 0.05).

### 3.3. Cardiometabolic Disease Risk Factors

**Figure 2:**
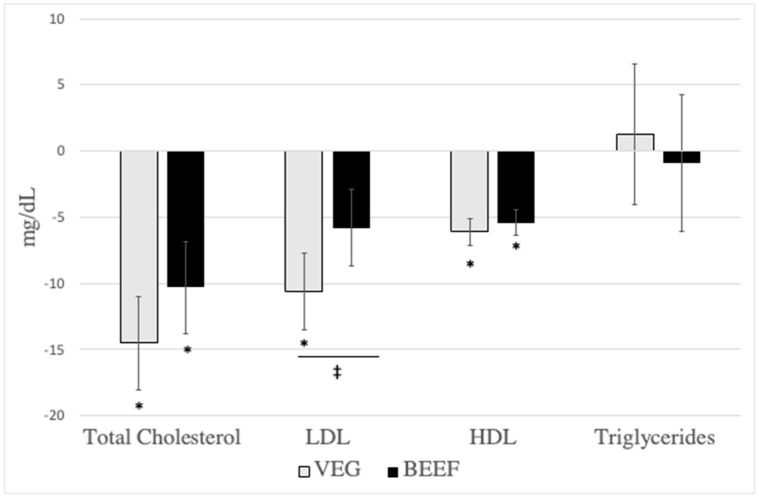
Effects of diet on lipids post-pre changes (N = 41). VEG (vegetarian healthy dietary patten), BEEF (beef healthy dietary pattern), LDL (low-density lipoprotein), HDL (high-density lipoprotein). All outcomes reported were from serum samples. Results are presented as LS means ± SE (N = 41). * Significant change over time, *p* < 0.05. ^‡^ Significant difference between VEG and BEEF (time × diet, *p* < 0.05).

**Figure 3:**
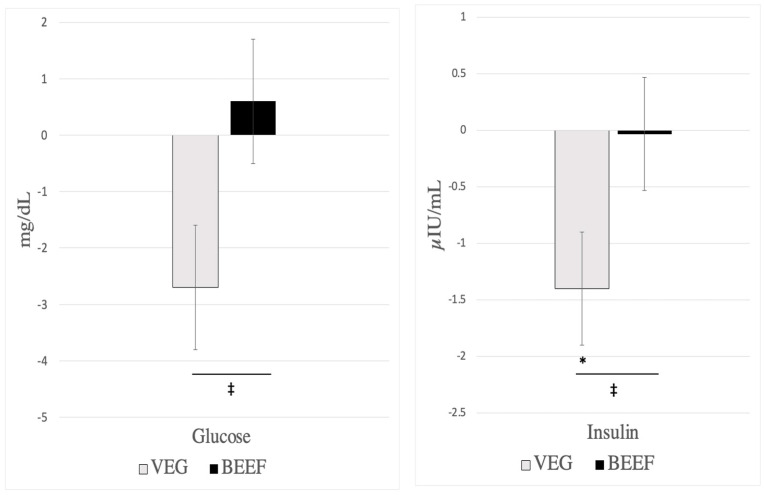
Effects of diet on glucose and insulin post-pre changes (N = 41). VEG (vegetarian healthy dietary patten), BEEF (beef healthy dietary pattern). All outcomes reported were from serum samples. Results are presented as LS means ± SE (N = 41). * Significant change over time, *p* < 0.05. ^‡^ Significant difference between VEG and BEEF (time × diet, *p* < 0.05).

Cardiometabolic disease risk factor measurements did not differ chronologically between baseline 1 and baseline 2 or experimentally before participants consumed the VEG vs. BEEF interventions (Table 3 and Table S3). Consumption of the VEG HDP, but not the BEEF HDP reduced LDL (−10.6 ± 3.0 vs. −5.8 ± 2.9 mg/dL, respectively, I×T *p* = 0.036), insulin (−1.4 ± 0.5 vs. −0.04 ± 0.5 µIU/mL, respectively, I×T *p* = 0.020), and glucose (−2.7 ± 1.1 vs 0.6 ± 1.1 mg/dL, respectively, I×T *p* = 0.001), Table 3, Figure 2 and Figure 3). Independent of VEG and BEEF interventions, HDP-induced reductions were observed for total cholesterol, HDL, dense LDL IV, buoyant HDL 2b, apolipoprotein A1, TC-to-HDL ratio, and systolic blood pressure (Table 3 and Table S3). No changes over time were found for apolipoprotein B, triglycerides, VLDL, lipoprotein(a), remnant lipoprotein, small, dense LDL III, diastolic blood pressure, waist circumference, hip circumference, and sagittal diameter (*p* > 0.05) (Table 3 and Table S3). Table S4 reports the unadjusted means ± SDs for all cardiometabolic disease risk factors, and Tables S5 and S6 report sex-specific unadjusted means ± SDs for all cardiometabolic disease risk factors for females and males, respectively. 

## 4. Discussion

This study provides novel insights into the effects of including lean, unprocessed beef in a HDP on cardiometabolic disease risk factors because few RCTs have compared a red meat diet intervention (void of other flesh foods) to a vegetarian diet intervention [12,13,23]. Our findings support that switching a U.S.-style vegetarian HDP to a U.S.-style omnivorous HDP by isoenergetically substituting predominately starchy vegetables and refined grains for lean, unprocessed beef significantly improved a number of CMD risk factors. These results are consistent with previous RCTs that compared a red meat diet to a vegetarian diet and found no differences in multiple CMD risk factors [12,13,23]. 

Systematic reviews and meta-analyses of RCTs support that unprocessed red meat and mixed unprocessed and processed red meats do not differentially affect changes in CMD risk factors [24,25,26,27]. However, the relative effect of red meat on CMD risk factors is influenced by the alternative food(s) substituted in the comparator diet [28]. Among limited research [29,30,31] aggregated in a meta-analysis [28], substituting red meat specifically for carbohydrates in dietary patterns did not influence changes in total or LDL cholesterol but favored reductions in triglycerides and higher HDL concentrations. The same meta-analysis [28] indicated that when red meat was substituted for high quality plant proteins (e.g., legumes, soy, and nuts), there were lesser reductions in total and LDL cholesterol and null effects on triglyceride and HDL concentrations [28]. Yet, when red meat was substituted for fish (e.g., fish, fatty fish, seafood), there were more favorable changes in total, LDL, and HDL cholesterol and neutral effects on triglycerides [28]. These relative effects [28], in combination with the findings from this study, suggest that lean, unprocessed red meat can be effectively included in an HDP that has beneficial effects on CMD risk factors. These results [28] underscore a complex conundrum regarding the health effects of consuming red meat [32]. 

Inconsistent findings indicate that intakes of red meat adversely contribute to CMD risk [32]. This debated diet-disease relationship is complicated by the types of red meat consumed (i.e., lean vs. non-lean; unprocessed vs. processed) and whether red meat is consumed within a relatively healthy or unhealthy dietary pattern. Red meat is often inconsistently described or categorized by researchers, which is problematic due to its heterogenous nutritional composition [33,34]. For instance, there is a wide variability in saturated fat and sodium contents among red meat products, for example, ranging from 1 g saturated fat and 48 mg sodium/serving of lean, unprocessed pork tenderloin to 39 g saturated fat and 775 mg sodium/serving of non-lean processed pork sausage [32]. Consistent with the results of the current RCT using U.S.-style HDPs, RCTs with DASH-style or Mediterranean-style HDPs that include lean and/or unprocessed red meat showed improvements in multiple CMD risk factors [7,8,9,10,11,23]. In contrast, findings among observational studies that assess cohorts who likely consume Western-style unhealthy dietary patterns report that high compared to low intakes of “total red and processed meats” increase the risk for T2DM and CVD incidence and mortality [35,36,37]. These inherently conflicting findings [7,8,9,10,11,35,36,37] regarding the effects of red meat intake on CMD risk factors, incidence, and mortality suggest that the type of red meat consumed (lean and/or unprocessed red meat vs. ‘total red and processed meats’) and the overall healthfulness of the dietary patterns (DASH or Mediterranean-style vs. Western-style) are likely contributing to these incongruent findings [38]. Clear and consistent definitions for meat-related terminology should be used in future observational and experimental research. Additionally, the healthfulness of dietary patterns with and without red meat should be considered when drawing conclusions about the effects that individual food items, such as lean, unprocessed red meat, have on health outcomes.

Our results show differential changes in serum glucose and insulin after the consumption of the VEG HDP vs. BEEF HDP, with these parameters trending down in the VEG HDP but not BEEF HDP. These results are partly consistent with previous research reporting reductions in either glucose or insulin after consuming a vegetarian dietary pattern [39,40]. Of note, the post-intervention insulin and glucose concentrations were not statistically different between VEG and BEEF. The diet-specific changes in glucose and insulin after consumption of VEG were unexpected because typically, dietary patterns that have a higher glycemic load and greater percent of energy from carbohydrates (e.g., the VEG HDP) would be expected to increase, rather than decrease, these metabolic outcomes [41]. Further, both VEG and BEEF dietary patterns were weight maintenance, and there were no changes in body mass post-intervention, which may have otherwise explained these findings [41]. Interestingly, these diet-specific changes in glucose and insulin were not supported by differential changes between diets in HOMA-IR, an indicator of insulin resistance [42]. The apparent lack of changes in glucose, insulin, and HOMA-IR after consuming the BEEF HDP complements previous findings reported in two meta-analyses [26,43]. 

The importance of assessing lipoprotein particle number and size is supported by emerging research [44,45,46] and the National Cholesterol Education Program Adult Treatment Panel III recommendations to improve CVD risk assessment [47]. Consuming the VEG or BEEF patterns decreased the number of total LDL and small, dense LDL IV particles. These are considered favorable improvements since small, dense LDL III and IV particles and a higher number of these particles impart the highest CVD risk due to easier and quicker penetration of the arterial endothelial lining. These findings complement existing research indicating that adopting HDPs with higher compared to lower intakes of red meat led to greater decreases in total LDL and either LDL III or LDL IV particles [44,45]. A higher number of HDL2b particles is considered more advantageous for cardiovascular health because they are the largest and most buoyant HDL subfraction and function to carry cholesterol back to the liver. After adopting either the VEG or BEEF patterns, we observed decreases in total HDL and HDL2b particles. These results are consistent with other research [44,46], after consuming diets with higher compared to lower red meat intake. 

Our study has several strengths. In addition to assessing both clinically meaningful CMD risk factors and lipoprotein particle numbers and sizes, we integrated strong experimental features (i.e., randomization, crossover design, researcher blinding, controlled feeding interventions, and double entry of data with crosscheck). While not objectively confirmed, participants self-reported ~89% adherence to both the VEG and BEEF HDP interventions. Adherence to the dietary patterns was crudely supported by higher blood urea nitrogen concentrations, consistent with higher total protein intake, during the BEEF compared to the VEG intervention. The observation that improvements in participants’ CMD risk factors after the first HDP intervention (chronologically) were reversed after the dietary washout period and returned after the second HDP intervention also supports dietary adherence, the concept of healthy dietary patterning [48], and encouragements for the general public to adopt and sustain HDPs for cardiometabolic health. The generalization of this study’s findings should be kept in context, as most of the included participants were Caucasian (85%). Only lean, unprocessed beef (e.g., ground beef, top sirloin, top round) was used in the BEEF intervention; therefore, these findings do not encompass non-lean and processed beef or non-beef sources of red meat (i.e., pork, lamb, veal, venison, goat). 

## 5. Conclusions

Consistent with recommendations from the Dietary Guidelines for Americans [4], results from this crossover randomized controlled feeding trial indicate that adopting a U.S.-style healthy dietary pattern that is either lacto-ovo vegetarian or omnivorous with lean, unprocessed beef results in short-term improvements in multiple cardiometabolic disease risk factors among adults classified as overweight or moderately obese.

## Figures and Tables

**Table 1:** Prescribed daily dietary intakes of the U.S.-style healthy dietary pattern interventions ^1^.

	VEG	BEEF
Energy (kcal/d)	2546 ± 150	2436 ± 79
Total Fat (g/d)	84 ± 13	91 ± 11
Total Carbohydrate (g/d)	361 ± 30	286 ± 28 ^a^
Total Protein (g/d)	105 ± 15	134 ± 9 ^a^
Animal Protein (g/d)	36 ± 12	81 ± 11 ^a^
Vegetable Protein (g/d)	69 ± 15	54 ± 10 ^a^
Cholesterol (mg/d)	200 ± 170	325 ± 158 ^a^
Total Saturated Fatty Acids (SFA) (g/d)	22 ± 5	25 ± 4 ^a^
Total Monounsaturated Fatty Acids (MUFA) (g/d)	37 ± 6	40 ± 6 ^a^
Total Polyunsaturated Fatty Acids (PUFA) (g/d)	19 ± 5	17 ± 4 ^a^
Glucose (g/d)	20 ± 6	19 ± 5
Total Dietary Fiber (g/d)	50 ± 7	41 ± 5 ^a^
Soluble Dietary Fiber (g/d)	9 ± 1	7 ± 1 ^a^
Insoluble Dietary Fiber (g/d)	40 ± 6	34 ± 5 ^a^
% Energy from Fat	29 ± 3	33 ± 3 ^a^
% Energy from Carbohydrate	56 ± 5	46 ± 4 ^a^
% Energy from Protein	15 ± 2	22 ± 2 ^a^
% Energy from SFA	7 ± 2	9 ± 2 ^a^
% Energy from MUFA	12 ± 2	14 ± 2 ^a^
% Energy from PUFA	6 ± 1	6 ± 1
Added Sugars (by Available Carbohydrate) (g/d)	18 ± 7	18 ± 7
Available Carbohydrate (g/d)	311 ± 26	244 ± 25 ^a^
Glycemic Index (glucose reference)	52 ± 2	51 ± 2
Glycemic Index (bread reference)	74 ± 4	73 ± 3
Glycemic Load (glucose reference)	161 ± 13	125 ± 13 ^a^
Glycemic Load (bread reference)	230 ± 19	179 ± 19 ^a^
Magnesium (mg/d)	573 ± 85	516 ± 53 ^a^
Sodium (mg/d)	3758 ± 442	3180 ± 381 ^a^
Potassium (mg/d)	4556 ± 230	4488 ± 260

Vegetarian healthy dietary pattern (VEG); beef healthy dietary pattern (BEEF). ^1^ Prescribed dietary intakes were averaged across a 7-day menu cycle. Results are presented as unadjusted means ± SDs. ^a^ The difference between VEG and BEEF dietary patterns was assessed by a two-tailed paired samples *t*-test, *p* < 0.05.

**Table 2:** Fasting subject characteristics at baseline (N = 41).

Outcome	Baseline 1
Age at enrollment, y	40 ± 8.1
Female, n (%)	22 (55)
Caucasian, n (%)	35 (85)
BMI, kg/m^2^	29.6 ± 3.3
Total cholesterol, mg/dL	179.5 ± 4.0
LDL, mg/dL	121.5 ± 3.4
HDL, mg/dL	48.1 ± 1.1
Triglycerides, mg/dL	98.1 ± 5.8
Glucose, mg/dL	93.3 ± 1.0
Insulin, µIU/mL	8.1 ± 0.5
SBP/DBP, mmHg	115 ± 1.2/76 ± 1.0

Values are means ± SE. N = 41. Body mass index (BMI), low-density lipoprotein (LDL), high-density lipoprotein (HDL), systolic blood pressure (SBP), diastolic blood pressure (DBP).

**Table 3:** Cardiometabolic disease risk factor responses from consuming VEG vs. BEEF HDP for 5 weeks (N = 41).

Outcome	VEG HDP			BEEF HDP			*p* Values	
	Pre	Post	Change	Pre	Post	Change	Time	Time × Diet
Total cholesterol (mg/dL)	180.5 ± 4	166.0 ± 4	−14.5 ± 3.5 *	178.8 ± 4	168.5 ± 4	−10.3 ± 3.5 *	0.001	0.166
LDL (mg/dL)	120.9 ± 3.5	110.3 ± 3.4	−10.6 ± 2.9 *	119.4 ± 3.4	113.7 ± 3.4	−5.8 ± 2.9	0.005	0.036
Total LDL particles (nmol/L)	870.3 ± 21.8	792.9 ± 21.8	−77.4 ± 19.8 *	853 ± 21.7	791.5 ± 21.8	−61.5 ± 19.7 *	<0.001	0.366
Dense LDL III (nmol/L)	288.3 ± 15.4	266.3 ± 15.3	−22 ± 16.1	273.5 ± 15.3	243.4 ± 15.4	−30.1 ± 16	0.063	0.636
Dense LDL IV (nmol/L)	77.6 ± 2.9	70.3 ± 2.9	−7.4 ± 3	75.8 ± 2.9	70.6 ± 2.9	−5.2 ± 3	0.010	0.577
HDL (mg/dL)	48.1 ± 1.2	42 ± 1.2	−6.1 ± 1 *	47.4 ± 1.2	42.1 ± 1.2	−5.4 ± 1 *	<0.001	0.415
Total HDL particles (nmol/L)	7036.8 ± 88.5	6687.3 ± 88.5	−349.5 ± 95 *	6998.7 ± 88.4	6663 ± 88.4	−335.7 ± 94.6 *	<0.001	0.916
Buoyant HDL2b (nmol/L)	2135 ± 59	1889.3 ± 58.8	−245.6 ± 51.6 *	2128 ± 58.7	1863.1 ± 58.9	−264.9 ± 51.1 *	<0.001	0.657
Apolipoprotein B (mg/dL)	88.5 ± 2.2	84.3 ± 2.2	−4.2 ± 2	87 ± 2.2	85.1 ± 2.2	−1.8 ± 2	0.100	0.158
Apolipoprotein A1(mg/dL)	135.7 ± 2.4	120.7 ± 2.4	−15.1 ± 2.2 *	134.5 ± 2.4	121.3 ± 2.4	−13.2 ± 2.2 *	<0.001	0.424
Triglycerides (mg/dL)	103.6 ± 5.7	104.9 ± 5.7	1.3 ± 5.3	98.3 ± 5.7	97.4 ± 5.7	−0.9 ± 5.2	0.967	0.681
Glucose (mg/dL)	94.7 ± 1.1	92.1 ± 1.1	−2.7 ± 1.1	93.3 ± 1.1	93.9 ± 1.1	0.6 ± 1.1	0.317	0.001
Insulin (µIU/mL)	9.2 ± 0.7	7.8 ± 0.7	−1.4 ± 0.5 *	7.8 ± 0.7	7.7 ± 0.7	−0.1 ± 0.5	0.100	0.020
HOMA-IR	2.4 ± 0.4	1.8 ± 0.5	−0.6 ± 0.5	1.8 ± 0.4	1.9 ± 0.5	0.1 ± 0.5	0.618	0.140
SBP (mmHg)	114.9 ± 1.2	112.2 ± 1.2	−2.7 ± 1.2	114 ± 1.2	112.5 ± 1.2	−1.5 ± 1.2	0.044	0.265
DBP (mmHg)	75.8 ± 1.1	74.4 ± 1.1	−1.4 ± 0.9	76.2 ± 1.1	74.4 ± 1.1	−1.8 ± 0.9	0.065	0.634

* Significant change from pre to post within intervention (*p* < 0.05). Vegetarian healthy dietary pattern (VEG); beef healthy dietary pattern (BEEF); healthy dietary pattern (HDP); low-density lipoprotein (LDL); high-density lipoprotein (HDL); homeostatic model assessment for insulin (HOMA-IR); systolic blood pressure (SBP); diastolic blood pressure (DBP). Data are reported as least squared means ± SE. Data were analyzed using a doubly repeated-measures ANOVA adjusted for age, sex, and body mass at each time point.

## Data Availability

The data supporting this project’s findings are available from the corresponding author, W.W.C., upon reasonable request.

## Supplementary Materials

The following supporting information can be downloaded at: https://www.mdpi.com/article/10.3390/nu16152542/s1; Table S1: Seven-Day Menu for BEEF and VEG Healthy Dietary Pattern (HDP); Table S2: Participant Dietary Satisfaction Questionnaire; Table S3: Cardiometabolic disease risk factor responses from consuming VEG vs. BEEF for 5 weeks (n = 41); Table S4: Unadjusted means and SD at each time point of VEG and BEEF (n = 41); Table S5: Unadjusted mean ± SD at each time point of VEG and BEEF for females (n = 22); Table S6: Unadjusted mean ± SD at each time point of VEG and BEEF for males (n = 19).
