# Supplementary material for: Healthy Dietary Patterns with and without Meat Improved Cardiometabolic Disease Risk Factors in Adults: A Randomized Crossover Controlled Feeding Trial
Source: Nutrients. 2024 Aug 3;16(15):2542. doi: 10.3390/nu16152542 (PMC11313868; doi:10.3390/nu16152542)
Supplement: Supplementary file 1 [file nutrients-16-02542-s001.zip › nutrients-3102021-supplementary.pdf]

Supplemental Materials

Healthy Dietary Patterns with and without Meat Improved Cardiometabolic Disease Risk Factors in Adults: A Randomized Crossover Controlled Feeding Trial

Erica R Hill, Yu Wang, Eric M Davis and Wayne W Campbell *

Department of Nutrition Science, Purdue University, West Lafayette, IN 47907, USA
* Correspondence: campbeww@purdue.edu; Tel.: +1-765-494-8236

Supplemental Table S1: Seven-Day Menu for BEEF and VEG Healthy Dietary Pattern (HDP)

Supplemental Table S2: Participant Dietary Satisfaction Questionnaire

Supplemental Table S3: Cardiometabolic disease risk factor responses from consuming VEG vs. BEEF for 5 weeks (n=41)

Supplemental Table S4: Unadjusted means and SD at each time point of VEG and BEEF (n=41)

Supplemental Table S5: Unadjusted mean ± SD at each time point of VEG and BEEF for females (n= 22)

Supplemental Table S6: Unadjusted mean ± SD at each time point of VEG and BEEF for males (n= 19)

Supplemental Table S1: Seven-Day Menu for BEEF and VEG Healthy Dietary Pattern (HDP)

Day	Meal	BEEF HDP	VEG HDP
**Day 1**	Breakfast	eggs, whole, raw<br>green pepper - sweet, raw<br>peppers, sweet red, raw<br>spinach, raw<br>tortilla, white flour, commercial type, plain<br>raspberries, frozen, unsweetened	eggs, whole, raw<br>green pepper - sweet, raw<br>peppers, sweet red, raw<br>spinach, raw<br>tortilla, white flour, commercial type, plain<br>raspberries, frozen, unsweetened
	Lunch	English muffin, whole wheat, plain or with seasoning<br>sauce, pizza<br>Mozzarella cheese, part skim milk, regular<br>almonds, roasted, oil roasted, salted<br>peanuts, roasted, oil roasted, salted<br>corn, yellow, cooked from frozen, whole kernel<br>ground beef or hamburger, 10% fat (90% lean meat)	English muffin, whole wheat, plain or with seasoning<br>sauce, pizza<br>Mozzarella cheese, part skim milk, regular<br>almonds, roasted, oil roasted, salted<br>peanuts, roasted, oil roasted, salted<br>corn, yellow, cooked from frozen, whole kernel
	Dinner	asparagus, raw<br>rice, white, instant (precooked)<br>milk, 1% fat or lowfat<br>oil, olive<br>tofu (soybean curd), silken, cooked, regular<br>green peas, cooked from frozen<br>lentils, cooked from dried<br>steak - beef, sirloin, no visible fat eaten	asparagus, raw<br>rice, white, instant (precooked)<br>milk, 1% fat or lowfat<br>oil, olive<br>tofu (soybean curd), silken, cooked, regular<br>green peas, cooked from frozen<br>lentils, cooked from dried
	Snack	apple, fresh, with skin<br>carrots, raw<br>celery, raw<br>dressing for salads, Kraft Free Ranch fat free	apple, fresh, with skin<br>carrots, raw<br>celery, raw<br>dressing for salads, Kraft Free Ranch fat free

Supplemental Table S1 Continued: Seven-Day Menu for BEEF and VEG Healthy Dietary Pattern (HDP)

Day	Meal	BEEF HDP	VEG HDP
Day 2	Breakfast	English muffin, whole wheat, plain or with seasoning<br>peanut butter, regular, with salt<br>milk, 1% fat or lowfat<br>apple, fresh, with skin	English muffin, whole wheat, plain or with seasoning<br>peanut butter, regular, with salt<br>milk, 1% fat or lowfat<br>apple, fresh, with skin
	Lunch	milk, 1% fat or lowfat<br>spaghetti sauce, without meat, commercial, plain, regular, regular<br>Parmesan cheese, dry (grated), regular<br>lettuce, romaine<br>carrots, raw<br>dressing for salads, Italian, commercial (include clear and creamy), regular<br>spaghetti noodles, white<br>ground beef or hamburger, 10% fat (90% lean meat)	milk, 1% fat or lowfat<br>spaghetti sauce, without meat, commercial, plain, regular, regular<br>Parmesan cheese, dry (grated), regular<br>lettuce, romaine<br>carrots, raw<br>dressing for salads, Italian, commercial (include clear and creamy), regular<br>spaghetti noodles, white<br>green peas, cooked from frozen
	Dinner	oil, olive<br>rolls, whole wheat<br>butter, regular, salted<br>peanuts, roasted, oil roasted, salted<br>soy sauce, regular<br>couscous, dry<br>beans, great northern, canned - drained, regular<br>orange, fresh<br>green or string beans, cooked from frozen<br>steak - beef, sirloin, no visible fat eaten	oil, olive<br>rolls, whole wheat<br>butter, regular, salted<br>peanuts, roasted, oil roasted, salted<br>soy sauce, regular<br>couscous, dry<br>beans, great northern, canned - drained, regular<br>orange, fresh<br>green or string beans, cooked from frozen
	Snack	chips - snack type, pita, regular<br>hummus (chickpea dip)<br>milk, 1% fat or lowfat<br>almonds, roasted, oil roasted, salted	chips - snack type, pita, regular<br>hummus (chickpea dip)<br>milk, 1% fat or lowfat<br>almonds, roasted, oil roasted, salted

Supplemental Table S1 Continued: Seven-Day Menu for BEEF and VEG Healthy Dietary Pattern (HDP)

Day	Meal	BEEF HDP	VEG HDP
Day 3	Breakfast	cereal, ready-to-eat, Grape-Nuts (Post)	cereal, ready-to-eat, Grape-Nuts (Post)
		yogurt, Dannon Oikos Greek Nonfat Yogurt - fruit flavors	yogurt, Dannon Oikos Greek Nonfat Yogurt - fruit flavors
		juice or flavored drink, apple, juice or cider, purchased ready-to-drink, unfortified	juice or flavored drink, apple, juice or cider, purchased ready-to-drink, unfortified
		blueberries, frozen, sweetened	blueberries, frozen, unsweetened
	Lunch	lettuce, romaine	lettuce, romaine
		eggs, whole, cooked	eggs, whole, cooked
		Cheddar cheese, natural	Cheddar cheese, natural
		dressing for salads, Italian, commercial (include clear and creamy), regular	dressing for salads, Italian, commercial (include clear and creamy), regular
		potato, baked, plain, skin eaten	potato, baked, plain, skin eaten
		butter, regular, salted	butter, regular, salted
		croutons, commercial, plain	croutons, commercial, plain
		beef, steak - beef, round, top, no visible fat eaten	
	Dinner	milk, 1% fat or lowfat	milk, 1% fat or lowfat
		oil, olive	oil, olive
		tortilla, white flour, commercial type, plain	tortilla, white flour, commercial type, plain
		tomato, paste	tomato, paste
		onion, white, yellow or red, raw	onion, white, yellow or red, raw
		spices, chili powder	spices, chili powder
		cumin, seed	cumin, seed
		lentils, cooked from dried	lentils, cooked from dried
		salsa, commercial	salsa, commercial
		ground beef or hamburger, 10% fat (90% lean meat)	lima beans
	Snack	wheat bread, whole wheat, regular, commercial	wheat bread, whole wheat, regular, commercial
		butter, regular, salted	butter, regular, salted
		celery, raw	celery, raw
		carrots, raw	carrots, raw

Supplemental Table S1 Continued: Seven-Day Menu for BEEF and VEG Healthy Dietary Pattern (HDP)

Day	Meal	BEEF HDP	VEG HDP
Day 4	Breakfast	cereal, ready-to-eat, Cheerios (General Mills)	cereal, ready-to-eat, Cheerios (General Mills)
		milk, 1% fat or lowfat	milk, 1% fat or lowfat
		banana, fresh or ripe	banana, fresh or ripe
		juice or flavored drink, apple, juice or cider, purchased ready-to-drink, unfortified	juice or flavored drink, apple, juice or cider, purchased ready-to-drink, unfortified
	Lunch	chips - snack type, Fritos Corn Chips - Original	chips - snack type, Fritos Corn Chips - Original
		ingredient, taco seasoning mix - dry mix	ingredient, taco seasoning mix - dry mix
		lettuce, romaine	lettuce, romaine
		refried beans, canned, regular	refried beans, canned, regular
		salsa, commercial	salsa, commercial
		corn, yellow, cooked from frozen, whole kernel	corn, yellow, cooked from frozen, whole kernel
		peppers, sweet red, raw	peppers, sweet red, raw
		oil, olive	oil, olive
		ground beef or hamburger, 10% fat (90% lean meat)	
	Dinner	oil, olive	oil, olive
		chickpeas, canned - drained, regular (drained and rinsed)	chickpeas, canned - drained, regular (drained and rinsed)
		celery, raw	celery, raw
		cucumber, raw, with peel	cucumber, raw, with peel
		juice or flavored drink, lemon juice, canned, bottled, or boxed	juice or flavored drink, lemon juice, canned, bottled, or boxed
		parsley, fresh	parsley, fresh
		mint, peppermint - fresh	mint, peppermint - fresh
		watercress, raw	watercress, raw
		rice, white, instant (precooked)	rice, white, instant (precooked)
		steak - beef, sirloin, no visible fat eaten	
	Snack	almonds, roasted, oil roasted, salted	almonds, roasted, oil roasted, salted
		wheat bread, whole wheat, regular, commercial	wheat bread, whole wheat, regular, commercial
		peanut butter, regular, with salt	peanut butter, regular, with salt

Supplemental Table S1 Continued: Seven-Day Menu for BEEF and VEG Healthy Dietary Pattern (HDP)

Day	Meal	BEEF HDP	VEG HDP
**Day 5**	Breakfast	eggs, whole, raw<br>green pepper - sweet, raw<br>peppers, sweet red, raw<br>spinach, raw<br>tortilla, white flour, commercial type, plain<br>juice or flavored drink, orange, juice, purchased ready-to-drink, fortified, with calcium and vitamin D	eggs, whole, raw<br>green pepper - sweet, raw<br>peppers, sweet red, raw<br>spinach, raw<br>tortilla, white flour, commercial type, plain<br>juice or flavored drink, orange, juice, purchased ready-to-drink, fortified, with calcium and vitamin D
	Lunch	soup, tomato, plain or cream of tomato, condensed can - undiluted, regular<br>wheat bread, whole wheat, regular, commercial<br>provolone cheese, natural<br>Swiss cheese, natural<br>butter, regular, salted<br>asparagus, raw<br>oil, olive<br>rice, white, instant (precooked)<br>ground beef or hamburger, 10% fat (90% lean meat)	soup, tomato, plain or cream of tomato, condensed can - undiluted, regular<br>wheat bread, whole wheat, regular, commercial<br>provolone cheese, natural<br>Swiss cheese, natural<br>butter, regular, salted<br>asparagus, raw<br>oil, olive<br>rice, white, instant (precooked)
	Dinner	milk, 1% fat or lowfat<br>onion, white, yellow or red, raw<br>tomato, raw<br>bouillon (broth), vegetable, regular<br>beans, black, canned - drained, regular<br>cumin, seed<br>paprika<br>nutmeg<br>garlic, powder<br>carrots, raw<br>celery, raw<br>almonds, roasted, oil roasted, salted<br>walnuts<br>peanuts, roasted, oil roasted, salted<br>almonds, roasted, oil roasted, salted<br>cranberries, dried (Craisins)<br>raisins, uncooked<br>ground beef or hamburger, 10% fat (90% lean meat)	milk, 1% fat or lowfat<br>onion, white, yellow or red, raw<br>tomato, raw<br>bouillon (broth), vegetable, regular<br>beans, black, canned - drained, regular<br>cumin, seed<br>paprika<br>nutmeg<br>garlic, powder<br>carrots, raw<br>celery, raw<br>almonds, roasted, oil roasted, salted<br>walnuts<br>peanuts, roasted, oil roasted, salted<br>almonds, roasted, oil roasted, salted<br>cranberries, dried (Craisins)<br>raisins, uncooked<br>sweet potato, raw
	Snack	chips - snack type, pita, regular<br>hummus (chickpea dip)<br>yogurt, Dannon Oikos Greek Nonfat Yogurt - fruit flavors<br>blueberries, frozen, unsweetened	chips - snack type, pita, regular<br>hummus (chickpea dip)<br>yogurt, Dannon Oikos Greek Nonfat Yogurt - fruit flavors<br>blueberries, frozen, unsweetened

Supplemental Table S1 Continued: Seven-Day Menu for BEEF and VEG Healthy Dietary Pattern (HDP)

Day	Meal	BEEF HDP	VEG HDP
**Day 6**	Breakfast	English muffin, whole wheat, plain or with seasoning<br>milk, 1% fat or lowfat<br>grapes, fresh<br>butter, regular, salted<br>jelly, regular	English muffin, whole wheat, plain or with seasoning<br>milk, 1% fat or lowfat<br>grapes, fresh<br>butter, regular, salted<br>jelly, regular
	Lunch	green peas, cooked from frozen<br>chickpeas, canned - drained, regular (drained and rinsed)<br>spinach, raw<br>feta cheese, regular<br>raisins, uncooked<br>juice or flavored drink, lemon juice, canned, bottled, or boxed<br>oil, olive<br>cumin, seed<br>spices, chili powder<br>corn, yellow, cooked from frozen, whole kernel<br>steak - beef, sirloin, no visible fat eaten	green peas, cooked from frozen<br>chickpeas, canned - drained, regular (drained and rinsed)<br>spinach, raw<br>feta cheese, regular<br>raisins, uncooked<br>juice or flavored drink, lemon juice, canned, bottled, or boxed<br>oil, olive<br>cumin, seed<br>spices, chili powder<br>corn, yellow, cooked from frozen, whole kernel<br>pita bread, white
	Dinner	couscous, dry<br>beans, great northern, canned - drained, regular<br>tortilla, white flour, commercial type, plain<br>carrots, raw<br>onion, white, yellow or red, raw<br>peppers, sweet red, raw<br>sunflower seeds, roasted, dry roasted, salted<br>spinach, raw<br>cottage cheese, nonfat<br>yogurt, Dannon Oikos Greek Nonfat Yogurt - plain<br>ginger (ground)<br>steak - beef, sirloin, no visible fat eaten	couscous, dry<br>beans, great northern, canned - drained, regular<br>tortilla, white flour, commercial type, plain<br>carrots, raw<br>onion, white, yellow or red, raw<br>peppers, sweet red, raw<br>sunflower seeds, roasted, dry roasted, salted<br>spinach, raw<br>cottage cheese, nonfat<br>yogurt, Dannon Oikos Greek Nonfat Yogurt - plain<br>ginger (ground)
	Snack	wheat bread, whole wheat, regular, commercial<br>butter, regular, salted<br>milk, 1% fat or lowfat<br>almonds, roasted, oil roasted, salted	wheat bread, whole wheat, regular, commercial<br>butter, regular, salted<br>milk, 1% fat or lowfat<br>almonds, roasted, oil roasted, salted

Supplemental Table S1 Continued: Seven-Day Menu for BEEF and VEG Healthy Dietary Pattern (HDP)

Day	Meal	BEEF HDP	VEG HDP
Day 7	Breakfast	cereal, ready-to-eat, Grape-Nuts (Post)	cereal, ready-to-eat, Grape-Nuts (Post)
		yogurt, Dannon Oikos Greek Nonfat Yogurt - fruit flavors	yogurt, Dannon Oikos Greek Nonfat Yogurt - fruit flavors
		juice or flavored drink, apple, juice or cider, purchased ready-to-drink, unfortified	juice or flavored drink, apple, juice or cider, purchased ready-to-drink, unfortified
		raspberries, frozen, unsweetened	raspberries, frozen, unsweetened
	Lunch	potato, baked, plain, skin eaten	potato, baked, plain, skin eaten
		butter, regular, salted	butter, regular, salted
		spaghetti noodles, white, cooked in salted water	spaghetti noodles, white, cooked in salted water
		tomato, raw	tomato, raw
		oil, olive	oil, olive
		basil, fresh	basil, fresh
		juice or flavored drink, lemon juice, canned, bottled, or boxed	juice or flavored drink, lemon juice, canned, bottled, or boxed
		olives, black	olives, black
		ground beef or hamburger, 10% fat (90% lean meat)	
	Dinner	milk, 1% fat or lowfat	milk, 1% fat or lowfat
		oil, olive	oil, olive
		tortilla, white flour, commercial type, plain	tortilla, white flour, commercial type, plain
		tomato, paste	tomato, paste
		onion, white, yellow or red, raw	onion, white, yellow or red, raw
		spices, chili powder	spices, chili powder
		cumin, seed	cumin, seed
		salsa, commercial	salsa, commercial
		beans, black, canned - drained, regular	beans, black, canned - drained, regular
		corn, yellow, cooked from frozen, whole kernel	corn, yellow, cooked from frozen, whole kernel
		ground beef or hamburger, 10% fat (90% lean meat)	
	Snack	wheat bread, whole wheat, regular, commercial	wheat bread, whole wheat, regular, commercial
		butter, regular, salted	butter, regular, salted
		carrots, raw	carrots, raw
		celery, raw	celery, raw
		dressing for salads, Kraft Ranch	dressing for salads, Kraft Ranch

Supplemental Table S2: Participant Dietary Satisfaction Questionnaire

Please respond to how much you agree with the following statements about the eating pattern (the collection of foods and beverages) that was provided to you by our lab for the past 5 weeks.

	Strongly disagree	Disagree	Slightly disagree	Slightly agree	Agree	Strongly agree
This eating pattern was satisfying.	1	2	3	4	5	6
My friends and family members would enjoy eating these foods.	1	2	3	4	5	6
My health and well-being improved after eating these foods for 5 weeks.	1	2	3	4	5	6
Adopting this eating pattern would make social gatherings difficult.	1	2	3	4	5	6
I did not enjoy this eating pattern.	1	2	3	4	5	6
I felt full after most meals.	1	2	3	4	5	6
I would not recommend others to adopt this eating pattern.	1	2	3	4	5	6
I enjoyed eating these meals.	1	2	3	4	5	6
I was still hungry after most meals.	1	2	3	4	5	6
Eating these foods made me feel good.	1	2	3	4	5	6
I would like to continue this eating pattern after the study is over.	1	2	3	4	5	6
I often craved salty foods.	1	2	3	4	5	6
This eating pattern was tasty.	1	2	3	4	5	6
My cravings for unhealthy foods were less frequent.	1	2	3	4	5	6
I would recommend this eating pattern to friends and family members.	1	2	3	4	5	6
I could see myself continuing to eat these foods after the study is over.	1	2	3	4	5	6
I often craved sugary foods.	1	2	3	4	5	6
I plan to continue this eating pattern after the study is over.	1	2	3	4	5	6
This eating pattern had a negative impact on my well-being.	1	2	3	4	5	6
I do not want to eat these foods any longer after the study is over.	1	2	3	4	5	6
It would be easy to grocery shop and cook these meals at home.	1	2	3	4	5	6
I could easily eat foods like this at restaurants or social events.	1	2	3	4	5	6
It was challenging eating these foods.	1	2	3	4	5	6

Supplemental Table S3: Cardiometabolic disease risk factor responses from consuming VEG vs. BEEF for 5 weeks (n=41)

	VEG			BEEF			P values	
**Outcome**	**Pre**	**Post**	**Change**	**Pre**	**Post**	**Change**	**Time**	**Time x Diet**
Sodium (mmol/L)	140.1 ± 0.2	140.4 ± 0.2	0.3 ± 0.2	140.5 ± 0.2	139.9 ± 0.2	-0.6 ± 0.2	0.404	0.006
Potassium (mmol/L)	4.3 ± 0	4.3 ± 0	-0.1 ± 0	4.3 ± 0	4.3 ± 0	0 ± 0	0.088	0.714
Chloride (mmol/L)	106.7 ± 0.3	107.2 ± 0.3	0.5 ± 0.3	107.3 ± 0.3	106.7 ± 0.3	-0.6 ± 0.3	0.755	0.003
CO2 (mmol/L)	22.5 ± 0.3	22.4 ± 0.3	-0.1 ± 0.3	22 ± 0.3	22.2 ± 0.3	0.2 ± 0.3	0.758	0.432
Glucose (mg/dL)	94.7 ± 1.1	92.1 ± 1.1	-2.7 ± 1.1	93.3 ± 1.1	93.9 ± 1.1	0.6 ± 1.1	0.317	0.001
BUN (mg/dL)	13.5 ± 0.5	12.8 ± 0.5	-0.7 ± 0.5	13.8 ± 0.5	14.8 ± 0.5	1 ± 0.5	0.752	0.001
Creatinine (mg/dL)	0.9 ± 0	0.8 ± 0	0 ± 0	0.9 ± 0	0.9 ± 0	0 ± 0	0.281	0.002
GFR (ml/min)	97.2 ± 1.5	100.3 ± 1.5	3.1 ± 1.3	97.9 ± 1.5	97.2 ± 1.5	-0.7 ± 1.3	0.318	0.003
Calcium (mg/dL)	9.1 ± 0.1	9.1 ± 0.1	0 ± 0.1	9.1 ± 0.1	9.2 ± 0.1	0 ± 0.1	0.745	0.484
Bilirubin (mg/dL)	0.5 ± 0	0.5 ± 0	0 ± 0	0.5 ± 0	0.5 ± 0	0 ± 0	0.287	0.194
Total Protein (g/dL)	6.7 ± 0.1	6.6 ± 0.1	-0.1 ± 0.1	6.7 ± 0.1	6.7 ± 0.1	0 ± 0.1	0.764	0.200
Albumin (g/dL)	4 ± 0	4 ± 0	0 ± 0	4 ± 0	4 ± 0	0 ± 0	0.767	0.996
ALP (U/L)	67.1 ± 2.6	66.9 ± 2.6	-0.3 ± 1.5	65.6 ± 2.6	66 ± 2.6	0.5 ± 1.5	0.943	0.573
ALT (U/L)	25.1 ± 2.2	23.9 ± 2.2	-1.2 ± 2.1	22.7 ± 2.2	24.8 ± 2.2	2.1 ± 2.1	0.785	0.216
AST (U/L)	20.8 ± 1.2	20.9 ± 1.2	0.1 ± 1.5	21.2 ± 1.2	22.3 ± 1.2	1.1 ± 1.5	0.612	0.636
VLDL Particles (nmol/L)	55.7 ± 4.1	62.1 ± 4.1	6.4 ± 4.5	55.2 ± 4.1	55.7 ± 4.1	0.4 ± 4.4	0.384	0.193
Total LDL Particles (nmol/L)	870.3 ± 21.8	792.9 ± 21.8	-77.4 ± 19.8	853 ± 21.7	791.5 ± 21.8	-61.5 ± 19.7	0.000	0.366
Non-HDL Particles (nmol/L)	926.1 ± 23.1	855.2 ± 23	-70.9 ± 21.4	908.2 ± 23	846.9 ± 23	-61.3 ± 21.3	0.001	0.625
Remnant Lipoprotein Particles (nmol/L)	115.3 ± 4.3	114.7 ± 4.3	-0.7 ± 4.5	112.1 ± 4.3	115.4 ± 4.3	3.3 ± 4.5	0.746	0.377
LDL III Particles (nmol/L)	288.3 ± 15.4	266.3 ± 15.3	-22 ± 16.1	273.5 ± 15.3	243.4 ± 15.4	-30.1 ± 16	0.063	0.636
LDL IV Particles (nmol/L)	77.6 ± 2.9	70.3 ± 2.9	-7.4 ± 3	75.8 ± 2.9	70.6 ± 2.9	-5.2 ± 3	0.010	0.577
Total HDL Particles (nmol/L)	7036.8 ± 88.5	6687.3 ± 88.5	-349.5 ± 95	6998.7 ± 88.4	6663 ± 88.4	-335.7 ± 94.6	<.0001	0.916
Buoyant HDL2b (nmol/L)	2135 ± 59	1889.3 ± 58.8	-245.6 ± 51.6	2128 ± 58.7	1863.1 ± 58.9	-264.9 ± 51.1	<.0001	0.657
Total Cholesterol (mg/dL)	180.5 ± 4	166 ± 4	-14.5 ± 3.5	178.8 ± 4	168.5 ± 4	-10.3 ± 3.5	0.001	0.166

Table S3 continued

Outcome	VEG Pre	VEG Post	VEG Change	BEEF Pre	BEEF Post	BEEF Change	P values Time	P values Time x Diet
Triglycerides (mg/dL)	103.6 ± 5.7	104.9 ± 5.7	1.3 ± 5.3	98.3 ± 5.7	97.4 ± 5.7	-0.9 ± 5.2	0.967	0.681
HDL (mg/dL)	48.1 ± 1.2	42 ± 1.2	-6.1 ± 1	47.4 ± 1.2	42.1 ± 1.2	-5.4 ± 1	<.0001	0.415
LDL (mg/dL)	120.9 ± 3.5	110.3 ± 3.4	-10.6 ± 2.9	119.4 ± 3.4	113.7 ± 3.4	-5.8 ± 2.9	0.005	0.036
Non-HDL (mg/dL)	131.8 ± 3.5	124.1 ± 3.5	-7.7 ± 3.3	130.9 ± 3.5	125.7 ± 3.5	-5.2 ± 3.3	0.043	0.366
Total Cholesterol:HDL (mg/dL)	3.8 ± 0.1	4 ± 0.1	0.2 ± 0.1	3.9 ± 0.1	4.1 ± 0.1	0.2 ± 0.1	0.008	0.838
Insulin (µIU/mL)	9.2 ± 0.7	7.8 ± 0.7	-1.4 ± 0.5	7.8 ± 0.7	7.7 ± 0.7	0 ± 0.5	0.100	0.020
Log CRP	0.5 ± 0.2	0.6 ± 0.2	0.1 ± 0.2	0.6 ± 0.2	0.7 ± 0.2	0.1 ± 0.2	0.559	0.852
Lipoprotein A (mg/dL)	27.8 ± 4.1	28.5 ± 4	0.7 ± 2.3	28.1 ± 4	31.1 ± 4	2.9 ± 2.2	0.421	0.078
Apolipoprotein B (mg/dL)	88.5 ± 2.2	84.3 ± 2.2	-4.2 ± 2	87 ± 2.2	85.1 ± 2.2	-1.8 ± 2	0.100	0.158
Apolipoprotein A1 (mg/dL)	135.7 ± 2.4	120.7 ± 2.4	-15.1 ± 2.2	134.5 ± 2.4	121.3 ± 2.4	-13.2 ± 2.2	<.0001	0.424
Homocysteine (µmol/L)	8.6 ± 0.3	8.4 ± 0.3	-0.2 ± 0.2	8.4 ± 0.3	8.3 ± 0.3	-0.1 ± 0.2	0.439	0.502
SBP (mmHg)	114.9 ± 1.2	112.2 ± 1.2	-2.7 ± 1.2	114 ± 1.2	112.5 ± 1.2	-1.5 ± 1.2	0.044	0.265
DBP (mmHg)	75.8 ± 1.1	74.4 ± 1.1	-1.4 ± 0.9	76.2 ± 1.1	74.4 ± 1.1	-1.8 ± 0.9	0.065	0.634
Waist Circumference (cm)	101.8 ± 0.8	102.6 ± 0.8	0.8 ± 0.4	102.1 ± 0.8	102.4 ± 0.8	0.4 ± 0.4	0.139	0.286
Hip Circumference (cm)	109 ± 0.6	109.7 ± 0.6	0.7 ± 0.4	109.3 ± 0.6	109.6 ± 0.6	0.3 ± 0.4	0.147	0.243
Sagittal Abdominal Diameter (cm)	23.8 ± 0.3	23.9 ± 0.2	0.1 ± 0.2	23.9 ± 0.2	23.8 ± 0.3	-0.1 ± 0.2	0.803	0.113

Supplemental Table S4: Unadjusted means and SD at each time point of VEG and BEEF (n=41)

	VEG						BEEF					
	Baseline		Post		Change		Baseline		Post		Change	
**Outcome**	mean	SD	mean	SD	mean	SD	mean	SD	mean	SD	mean	SD
BMI (kg/m^2)	29.9	3.2	29.3	3.1	-0.60	0.5	29.7	3.2	29.2	3.1	-0.40	0.6
Sodium (mmol/L)	140.1	1.6	140.3	1.8	0.24	1.6	140.4	1.8	139.8	1.7	-0.60	1.3
Potassium (mmol/L)	4.3	0.2	4.3	0.2	-0.05	0.2	4.3	0.2	4.3	0.2	-0.04	0.2
Chloride (mmol/L)	106.7	1.6	107.1	1.7	0.43	1.5	107.2	1.7	106.6	1.7	-0.60	1.6
CO2 (mmol/L)	22.4	1.8	22.4	2.2	0.01	2.1	21.9	2.3	22.2	2.0	0.32	2.1
Glucose (mg/dL)	94.6	8.1	91.9	7.5	-2.72	5.8	93.2	8.3	93.7	8.0	0.46	6.3
BUN (mg/dL)	13.3	3.4	12.7	3.3	-0.61	2.2	13.7	4.1	14.6	3.7	0.93	3.1
Creatinine (mg/dL)	0.9	0.1	0.8	0.1	-0.03	0.1	0.9	0.1	0.9	0.1	0.01	0.1
GFR (ml/min)	97.1	13.5	100.4	10.9	3.29	7.3	97.7	13.2	97.0	13.2	-0.66	6.9
Calcium (mg/dL)	9.1	0.4	9.1	0.4	-0.02	0.4	9.2	0.4	9.2	0.4	0.03	0.4
Bilirubin (mg/dL)	0.5	0.2	0.5	0.2	0.05	0.1	0.5	0.2	0.5	0.3	0.01	0.2
Total Protein (g/dL)	6.7	0.3	6.6	0.3	-0.07	0.3	6.7	0.4	6.7	0.4	0.01	0.3
Albumin (g/dL)	4.0	0.2	4.0	0.2	0.01	0.2	4.0	0.3	4.0	0.2	0.00	0.2
APT (U/L)	67.4	18.7	66.9	19.6	-0.51	6.5	65.9	19.2	66.2	20.7	0.28	6.7
ALT (U/L)	24.9	14.1	22.5	12.3	-2.38	8.0	22.8	10.8	25.0	20.2	2.28	17.9
AST (U/L)	20.8	5.6	20.6	5.5	-0.18	3.4	21.3	7.2	22.2	11.7	0.96	13.0
VLDL Particles (nmol/L)	55.8	25.7	61.9	26.5	6.11	20.2	55.0	30.2	55.1	27.5	0.12	27.9
Total LDL Particles (nmol/L)	872.7	159.7	795.0	161.1	-77.76	101.9	854.6	155.9	792.2	147.9	-62.41	108.8
Non-HDL Particles (nmol/L)	928.5	167.9	857.0	169.5	-71.55	106.7	909.5	164.6	847.2	157.1	-62.28	121.1
Remnant Lipoprotein Particles (nmol/L)	115.8	27.3	114.8	24.4	-1.00	25.0	111.9	30.3	114.9	31.5	3.01	27.9
LDL III Particles (nmol/L)	285.6	118.0	261.1	98.7	-24.56	94.6	273.3	103.1	246.2	101.7	-27.10	104.1
LDL IV Particles (nmol/L)	78.3	15.7	71.0	20.6	-7.26	15.5	76.6	28.6	71.6	18.7	-4.96	19.8

Table S4 Continued

	VEG						BEEF					
	**Baseline**		**Post**		**Change**		**Baseline**		**Post**		**Change**	
**Outcome**	mean	SD	mean	SD	mean	SD	mean	SD	mean	SD	mean	SD
Total HDL Particles (nmol/L)	7036.8	493.4	6708.8	610.7	-328.04	518.1	7017.7	734.4	6686.7	616.0	-330.99	647.0
HDL2b (nmol/L)	2152.4	512.7	1915.6	476.9	-236.83	226.0	2148.2	589.1	1888.7	488.6	-259.46	263.0
Total Cholesterol (mg/dL)	181.0	29.8	166.8	30.5	-14.17	17.3	179.3	29.3	169.2	29.3	-10.15	15.8
Triglycerides (mg/dL)	104.2	39.8	104.3	33.1	0.10	25.8	98.2	40.5	96.5	39.9	-1.70	32.9
HDL (mg/dL)	48.4	10.4	42.5	9.4	-5.93	4.8	47.9	10.9	42.6	9.5	-5.29	4.9
LDL (mg/dL)	3.8	0.7	4.0	0.7	0.19	0.5	3.9	0.8	4.1	0.7	0.21	0.3
Non-HDL (mg/dL)	121.0	26.5	110.7	25.2	-10.28	15.9	119.6	24.6	113.9	24.3	-5.71	12.5
Total Cholesterol:HDL (mg/dL)	131.2	25.6	124.5	26.3	-7.68	16.2	131.1	25.1	125.6	24.7	-4.83	14.1
Insulin (μIU/mL)	9.3	5.4	7.7	4.5	-1.58	3.8	7.9	4.2	7.5	3.8	-0.39	2.7
CRP (mg/dL)	2.6	2.6	3.7	7.5	1.05	7.4	3.2	4.0	3.9	5.9	0.63	5.8
Log CRP	0.5	1.0	0.5	1.2	-0.01	0.9	0.7	1.0	0.7	1.1	0.01	0.9
Lipoprotein A (mg/dL)	27.1	30.4	29.4	33.2	2.24	6.9	28.2	32.7	32.1	36.6	3.92	8.2
Apolipoprotein B (mg/dL)	88.7	17.4	84.7	17.1	-3.94	10.2	87.1	16.0	85.4	15.1	-1.71	9.2
Apolipoprotein A1 (mg/dL)	136.2	20.0	121.5	18.6	-14.66	10.6	135.4	22.0	122.3	18.8	-13.04	13.1
Homocysteine (μmol/L)	8.5	2.1	8.3	2.1	-0.18	1.1	8.3	2.2	8.3	2.2	-0.07	1.2
SBP (mmHg)	114.5	9.5	111.9	9.7	-2.64	6.1	113.6	9.7	112.0	8.9	-1.59	6.1
DBP (mmHg)	76.0	7.9	74.4	8.2	-1.61	4.5	76.3	7.6	74.4	6.1	-1.99	4.6
Waist Circumference (cm)	102.5	10.8	101.8	10.9	-0.73	2.3	102.3	10.7	101.6	10.2	-0.62	2.0
Hip Circumference (cm)	110.0	9.4	109.4	9.3	-0.57	2.0	109.8	9.3	109.3	9.4	-0.52	1.7
Sagittal Abdominal Diameter (cm)	24.0	3.0	23.7	2.8	-0.29	0.9	23.9	2.7	23.6	2.4	-0.36	0.8

Supplemental Table S5: Unadjusted mean ± SD at each time point of VEG and BEEF for females (n= 22)

	VEG						BEEF					
	**Baseline**		**Post**		**Change**		**Baseline**		**Post**		**Change**	
**Outcome**	mean	SD	mean	SD	mean	SD	mean	SD	mean	SD	mean	SD
BMI (kg/m^2)	29.9	3.4	29.3	3.3	-0.56	0.4	29.9	3.4	29.4	3.4	-0.50	0.5
Sodium (mmol/L)	139.5	1.5	139.6	1.8	0.09	1.8	139.8	1.6	139.1	1.5	-0.70	1.3
Potassium (mmol/L)	4.3	0.2	4.3	0.2	-0.02	0.2	4.3	0.2	4.3	0.2	-0.05	0.2
Chloride (mmol/L)	106.5	1.6	106.7	1.5	0.20	1.5	107.0	1.8	106.6	1.8	-0.34	1.9
CO2 (mmol/L)	21.9	1.6	21.9	2.2	0.00	2.2	21.4	2.3	21.7	1.5	0.34	2.1
Glucose (mg/dL)	92.2	7.4	89.5	6.6	-2.73	6.0	90.9	8.5	91.6	8.8	0.73	7.9
BUN (mg/dL)	11.8	3.0	11.4	3.3	-0.39	2.0	12.6	4.5	13.0	2.9	0.48	3.1
Creatinine (mg/dL)	0.8	0.1	0.8	0.1	-0.02	0.0	0.8	0.1	0.8	0.1	0.00	0.1
GFR (ml/min)	96.2	12.9	98.6	11.0	2.41	6.6	96.1	12.4	96.4	12.6	0.30	7.1
Calcium (mg/dL)	9.1	0.4	9.1	0.6	0.03	0.4	9.2	0.5	9.2	0.5	0.07	0.3
Bilirubin (mg/dL)	0.4	0.2	0.4	0.1	0.04	0.1	0.5	0.2	0.5	0.2	0.00	0.2
Total Protein (g/dL)	6.7	0.4	6.6	0.3	-0.10	0.3	6.8	0.4	6.8	0.4	0.00	0.4
Albumin (g/dL)	4.0	0.2	4.0	0.2	-0.01	0.2	4.0	0.3	4.0	0.2	0.00	0.2
APT (U/L)	70.3	21.5	68.9	23.4	-1.39	7.6	68.6	21.8	67.7	22.9	-0.93	7.2
ALT (U/L)	19.8	10.8	18.6	10.6	-1.27	8.7	18.6	8.3	24.1	25.9	5.45	23.6
AST (U/L)	19.0	4.6	18.7	4.4	-0.32	3.9	20.4	7.9	22.4	14.4	2.05	17.2
VLDL Particles (nmol/L)	54.3	24.2	60.1	25.2	5.82	23.3	52.2	29.8	49.2	24.4	-3.05	26.9
Total LDL Particles (nmol/L)	896.0	159.2	825.9	184.8	-70.09	107.0	865.2	178.4	814.5	146.8	-50.75	119.7
Non-HDL Particles (nmol/L)	950.1	168.6	886.1	196.5	-64.00	117.4	917.3	188.0	863.7	158.9	-53.66	129.7
Remnant Lipoprotein Particles (nmol/L)	114.2	27.3	118.4	24.7	4.16	27.0	110.4	29.8	111.6	31.0	1.20	31.0
LDL III Particles (nmol/L)	273.0	118.4	247.0	93.5	-25.93	101.5	255.4	103.4	221.8	79.4	-33.64	101.9
LDL IV Particles (nmol/L)	86.1	13.4	79.8	23.1	-6.25	19.0	88.3	34.1	81.4	18.2	-6.91	24.5
Total HDL Particles (nmol/L)	7169.0	462.6	6848.6	597.0	-320.39	559.1	7256.3	729.5	6977.2	525.9	-279.07	710.9

Table S5 Continued:

	VEG						BEEF					
	Baseline		Post		Change		Baseline		Post		Change	
**Outcome**	mean	SD	mean	SD	mean	SD	mean	SD	mean	SD	mean	SD
HDL2b (nmol/L)	2423.0	524.3	2164.1	497.2	-258.98	270.2	2475.0	573.6	2169.5	476.9	-305.48	261.5
Total Cholesterol (mg/dL)	188.8	29.7	176.2	35.1	-12.61	17.2	185.3	33.0	176.5	27.4	-8.80	16.6
Triglycerides (mg/dL)	101.4	37.6	101.2	32.0	-0.25	21.5	98.4	42.4	88.5	35.7	-9.91	31.7
HDL (mg/dL)	53.5	10.9	47.1	9.6	-6.39	5.4	54.1	10.6	48.3	8.7	-5.89	5.1
LDL (mg/dL)	3.6	0.7	3.8	0.6	0.18	0.5	3.5	0.6	3.7	0.6	0.24	0.4
Non-HDL (mg/dL)	123.0	26.9	115.4	29.2	-7.61	14.7	120.6	26.0	117.2	23.6	-3.45	13.5
Total Cholesterol:HDL (mg/dL)	135.3	25.6	129.3	30.5	-5.98	17.1	130.8	27.8	128.2	23.6	-2.61	15.2
Insulin (μIU/mL)	9.8	6.2	7.7	3.9	-2.08	3.7	7.9	3.8	7.7	4.2	-0.19	2.6
CRP (mg/dL)	3.6	3.2	5.6	9.9	2.04	10.2	3.9	4.7	5.4	7.5	1.49	7.4
Log CRP	0.8	1.1	0.9	1.4	0.10	1.1	0.8	1.1	0.9	1.3	0.16	1.1
Lipoprotein A (mg/dL)	30.0	31.7	32.8	34.8	2.80	6.4	32.3	34.6	35.9	39.1	3.55	9.6
Apolipoprotein B (mg/dL)	91.9	18.9	88.7	19.9	-3.27	10.3	89.7	19.1	88.0	16.0	-1.66	11.0
Apolipoprotein A1 (mg/dL)	145.2	20.2	129.9	18.8	-15.30	11.8	147.8	20.3	132.6	17.3	-15.25	12.1
Homocysteine (μmol/L)	7.6	1.6	7.2	1.7	-0.40	0.9	7.4	1.9	7.3	1.7	-0.06	1.1
SBP (mmHg)	110.7	6.0	108.6	8.0	-2.09	5.5	109.7	7.7	109.2	9.5	-0.49	5.8
DBP (mmHg)	76.1	6.7	76.1	8.0	0.01	4.2	76.0	7.1	75.3	6.1	-0.73	3.4
Waist Circumference (cm)	101.4	10.3	100.7	10.7	-0.69	2.5	101.4	10.5	100.7	10.2	-0.68	2.0
Hip Circumference (cm)	112.8	9.4	112.2	9.2	-0.61	1.8	113.0	9.3	112.1	9.6	-0.90	1.5
Sagittal Abdominal Diameter (cm)	23.6	2.8	23.3	2.7	-0.30	0.7	23.5	2.6	23.2	2.4	-0.34	0.9

Supplemental Table S6: Unadjusted mean ± SD at each time point of VEG and BEEF for males (n= 19)

	VEG						BEEF					
	**Baseline**		**Post**		**Change**		**Baseline**		**Post**		**Change**	
**Outcome**	mean	SD	mean	SD	mean	SD	mean	SD	mean	SD	mean	SD
BMI (kg/m^2)	29.9	3.1	29.2	3.0	-0.64	0.6	29.4	3.0	29.1	2.9	-0.29	0.6
Sodium (mmol/L)	140.8	1.5	141.2	1.3	0.42	1.3	141.1	1.8	140.6	1.7	-0.47	1.3
Potassium (mmol/L)	4.3	0.2	4.2	0.2	-0.08	0.1	4.3	0.2	4.2	0.2	-0.03	0.3
Chloride (mmol/L)	106.9	1.5	107.6	1.8	0.68	1.6	107.6	1.6	106.7	1.7	-0.89	1.1
CO2 (mmol/L)	22.9	1.8	23.0	2.1	0.03	2.1	22.5	2.2	22.8	2.4	0.29	2.1
Glucose (mg/dL)	97.3	8.1	94.6	7.7	-2.71	5.9	95.9	7.3	96.0	6.4	0.16	4.2
BUN (mg/dL)	15.1	3.0	14.2	2.5	-0.87	2.5	15.0	3.3	16.4	3.9	1.45	3.3
Creatinine (mg/dL)	1.0	0.1	0.9	0.1	-0.04	0.1	0.9	0.1	1.0	0.1	0.02	0.1
GFR (ml/min)	98.0	14.4	102.3	10.8	4.32	8.2	99.6	14.1	97.8	14.2	-1.76	6.9
Calcium (mg/dL)	9.1	0.3	9.1	0.2	-0.07	0.4	9.1	0.2	9.1	0.2	-0.01	0.4
Bilirubin (mg/dL)	0.5	0.2	0.6	0.2	0.06	0.2	0.6	0.3	0.6	0.3	0.01	0.2
Total Protein (g/dL)	6.7	0.3	6.6	0.4	-0.04	0.3	6.6	0.3	6.6	0.3	0.02	0.3
Albumin (g/dL)	4.1	0.2	4.1	0.2	0.02	0.2	4.1	0.2	4.1	0.2	0.01	0.3
APT (U/L)	64.0	14.5	64.5	14.3	0.50	5.2	62.8	15.7	64.4	18.1	1.68	6.2
ALT (U/L)	30.7	15.6	27.0	12.7	-3.66	7.3	27.6	11.7	26.2	11.1	-1.39	7.5
AST (U/L)	22.8	6.0	22.8	6.0	-0.03	3.0	22.3	6.3	22.0	7.9	-0.29	6.0
VLDL Particles (nmol/L)	57.6	28.0	64.1	28.4	6.45	17.1	58.1	31.3	61.9	29.8	3.79	30.1
Total LDL Particles (nmol/L)	845.8	160.3	759.2	123.7	-86.63	100.7	842.4	128.8	766.4	148.9	-75.92	99.3
Non-HDL Particles (nmol/L)	903.6	168.2	823.3	128.6	-80.29	98.5	900.5	137.0	828.2	157.0	-72.26	116.7
Remnant Lipoprotein Particles (nmol/L)	117.6	28.0	110.6	24.0	-6.97	22.5	113.6	31.7	118.7	32.5	5.11	25.2
LDL III Particles (nmol/L)	300.3	119.0	277.4	104.6	-22.97	91.6	294.0	101.5	274.5	118.6	-19.53	111.6
LDL IV Particles (nmol/L)	69.2	13.3	60.8	10.7	-8.42	11.1	63.0	10.1	60.3	11.9	-2.71	13.5
Total HDL Particles (nmol/L)	6883.7	495.2	6546.8	601.0	-336.89	496.4	6741.4	653.8	6350.3	545.9	-391.11	597.3
HDL2b (nmol/L)	1839.0	265.2	1627.8	232.8	-211.18	173.0	1769.8	325.3	1563.6	239.4	-206.18	268.8

Table S6 Continued

Outcomes	VEG						BEEF					
	Baseline		Post		Change		Baseline		Post		Change	
	mean	SD	mean	SD	mean	SD	mean	SD	mean	SD	mean	SD
Total Cholesterol (mg/dL)	172.0	27.9	156.0	20.0	-15.97	18.3	172.3	23.2	160.6	29.8	-11.71	15.6
Triglycerides (mg/dL)	107.4	43.0	107.9	34.8	0.50	31.3	97.9	39.3	105.8	43.4	7.82	33.3
HDL (mg/dL)	42.5	5.6	37.1	5.5	-5.39	4.1	40.7	5.6	36.1	5.5	-4.61	4.8
LDL (mg/dL)	4.1	0.6	4.3	0.8	0.20	0.4	4.3	0.7	4.5	0.7	0.18	0.3
Non-HDL (mg/dL)	118.7	26.6	105.3	18.9	-13.37	17.5	118.3	23.5	110.0	25.1	-8.32	11.4
Total Cholesterol:HDL (mg/dL)	125.9	25.3	118.9	19.7	-9.94	15.9	131.5	22.2	122.5	26.3	-7.50	12.9
Insulin (μIU/mL)	8.7	4.4	7.7	5.3	-1.01	4.1	8.0	4.7	7.3	3.3	-0.62	2.9
CRP (mg/dL)	1.5	1.0	1.4	1.0	-0.11	0.8	2.5	3.0	2.1	2.4	-0.37	3.4
Log CRP	0.3	0.7	0.1	0.8	-0.13	0.5	0.6	0.9	0.4	0.9	-0.16	0.7
Lipoprotein A (mg/dL)	23.9	29.3	25.5	31.7	1.59	7.8	23.3	30.4	27.7	34.0	4.33	6.8
Apolipoprotein B (mg/dL)	84.9	15.1	80.2	12.0	-4.71	10.6	84.1	11.4	82.4	13.8	-1.76	7.2
Apolipoprotein A1 (mg/dL)	125.7	13.9	111.8	13.0	-13.92	9.7	120.9	13.5	110.4	12.4	-10.47	14.3
Homocysteine (μmol/L)	9.5	2.2	9.6	1.9	0.07	1.3	9.4	2.0	9.4	2.3	-0.08	1.4
SBP (mmHg)	119.13	10.90	115.82	10.28	-3.31	7.1	118.39	10.05	115.46	7.02	-2.93	6.5
DBP (mmHg)	75.82	9.41	72.24	8.24	-3.58	4.4	76.75	8.42	73.22	6.01	-3.53	5.5
Waist Circumference (cm)	103.87	11.61	103.09	11.35	-0.77	2.2	103.34	11.21	102.79	10.50	-0.55	2.0
Hip Circumference (cm)	106.55	8.31	106.03	8.37	-0.53	2.4	105.98	7.92	105.94	8.18	-0.05	1.9
Sagittal Abdominal Diameter (cm)	24.47	3.17	24.19	2.80	-0.28	1.1	24.39	2.93	24.00	2.39	-0.39	0.8
